# The Simple Cholestatic Complaints Score is a valid and quick patient‐reported outcome measure in primary sclerosing cholangitis

**DOI:** 10.1111/liv.14644

**Published:** 2020-10-28

**Authors:** Kim N. van Munster, Marcel G. W. Dijkgraaf, Sara van Gennep, Ulrich Beuers, Cyriel Y. Ponsioen

**Affiliations:** ^1^ Department of Gastroenterology and Hepatology Amsterdam University Medical Centers location AMC Amsterdam Gastroenterology & Metabolism Amsterdam The Netherlands; ^2^ Department of Epidemiology and Data Science Amsterdam University Medical Centers University of Amsterdam Amsterdam The Netherlands

**Keywords:** abdominal pain, burden, fatigue, patient‐reported outcome, pruritus, PSC, questionnaire, validation

## Abstract

**Background:**

Measuring symptoms and disease burden in patients with primary sclerosing cholangitis (PSC) is increasingly important for daily practice and clinical trials. The Simple Cholestatic Complaints Score (SCCS) is a four‐item questionnaire, that measures cholestatic symptoms (pruritus, fatigue, RUQ abdominal pain and fever) in PSC patients. The aim of this study was to evaluate reliability and validity of SCCS in a Dutch population.

**Methods:**

The study population consisted of 212 patients from the Dutch prospective PSC registry. Data were collected via digital surveys. Reliability was evaluated by internal consistency and reproducibility. Construct‐, criterion‐ and discriminant validity were determined. The ability to detect clinical change with SCCS was evaluated in patients who underwent endoscopic intervention. Simple Cholestatic Complaints Score collected by email and by a mobile application were compared.

**Results:**

A total of 153 patients completed the questionnaire. Internal consistency was moderate and increased to 0.71 after removal of the fever item. Test‐re‐test reproducibility was high (intraclass correlation coefficient = 0.96). Criterion validity was good (all > 0.82). Construct validity was in line with a priori hypothesized correlations in 80%. SCCS was able to differentiate between clinically different groups. There was no difference between inflammatory bowel disease (IBD) and non‐IBD patients. Simple Cholestatic Complaints Score was responsive to change after endoscopic intervention in successfully treated patients. Simple Cholestatic Complaints Score measurement by digital questionnaire and a mobile application was comparable.

**Conclusion:**

The SCCS is a valid instrument to measure cholestatic symptoms in PSC patients. Because of its quick and easy to use properties it is suitable for frequent monitoring of symptoms in clinical trials and daily practice.

**Abbreviations**
EQ‐5D: EuroQol‐5D
FIS: Fatigue Impact Scale
HrQoL: Health‐Related Quality of Life
IBD: Inflammatory Bowel Disease
ICC: Intraclass Correlation Coefficient
iPCQ: iMTA production loss cost questionnaire
LD: Large Duct
LDSI: Liver Disease Symptom Index
PRO: Patient‐Reported Outcome
PSC: Primary Sclerosing Cholangitis
RUQ: Right Upper Quadrant
SCCS: Short Cholestatic Complaints Score
SD: Small Duct
VAS: Visual Analogue Scale

**Lay Summary**

Patients with primary sclerosing cholangitis often suffer from cholestatic complaints such as itch, exhaustion and abdominal pain. It is important to measure the degree of complaints to monitor the effect of new therapies. In this study we have shown that the short cholestatic complaints score (SCCS) is able to accurately measure cholestatic complaints in PSC patients.

## 1 INTRODUCTION

Primary sclerosing cholangitis (PSC) is a rare cholestatic disease in which bile duct inflammation leads to destruction of the biliary tree.^1^ The disease has a strong association with inflammatory bowel disease, in particular with ulcerative colitis. Primary sclerosing cholangitis patients have an increased risk of developing cholangiocarcinoma, colon carcinoma and end‐stage liver disease. Most patients suffer from cholestatic symptoms, such as pruritus, fatigue and right upper quadrant (RUQ) abdominal pain. Improvement of symptoms is an important therapeutic goal, as these symptoms have major impact on quality of life.^2^ In the past years, new therapeutic options for the management of cholestatic pruritus have evolved.^3^, ^4^ Currently, there is a resurgent interest in developing new agents to treat this orphan disease. However, there is an apparent lack of available patient‐reported outcome (PRO) measurement tools in PSC.^5^ Recently, the PSC PRO, a questionnaire measuring health‐related quality of life (HrQoL) in PSC patients, was developed and validated.^6^ Primary sclerosing cholangitis PRO consists of 42 items and is divided into two modules, symptoms and impact of symptoms. Although this questionnaire is useful for very detailed measurement of HrQoL and disease burden, it is not suitable for daily practice or frequent issuing in clinical trials because of its complexity. Ideally, measurement of presence and/or change of cholestatic symptoms and their impact on daily life requires an easy and validated tool.^7^

Another PSC specific symptom score is the Simple Cholestatic Complaints Score (SCCS), which was first described in 1999.^8^ It is a four‐item questionnaire, containing questions about pruritus, fatigue, right upper quadrant abdominal pain and fever. It takes less than 1 minute to complete and has been applied in both clinical trials^8^, ^9^ and daily practice. However, the questionnaire has so far not been validated.

The aim of this study is to formally validate the SCCS for use in clinical trials in a Dutch PSC population.

## 2 MATERIALS AND METHODS

### 2.1 Simple Cholestatic Complaints Score

The SCCS (Table 1) contains four questions about degree of pruritus, fatigue, RUQ abdominal pain and fever in the past 7 days. The pruritus, fatigue and abdominal pain item have a score from 0 to 4, the fever item score is 0 or 1. The sum score, a sum of the four individual scores, ranges from 0 to 13.

**TABLE 1:** Scoring of the simple cholestatic complaints score

Score	Pruritus	Fatigue	RUQ‐A pain	Fever
0	No	No	No	No
1	Sometimes	Not able to do everything	Sometimes	Yes
2	Daily	Have to rest	Daily	
3	Wakes me up or I use antipruritic drugs	In bed more than half of the day	Wakes me up or I use painkillers	
4	Unbearable itch	All day in bed	Unbearable pain	

### 2.2 Study population, design and data collection

A cross sectional design was used for this study. Data were collected from August 2017 to November 2017. The study population consisted of patients of the EpiPSC 2 study, a large Dutch population‐based prospective registry.^10^ Diagnosis was based on EASL criteria, and all patients underwent careful case ascertainment on site. Small Duct PSC was defined as clinical and histological signs of PSC without cholangiographic changes. All patients received periodic digital surveys via email and/or a mobile application. Data were collected in a web‐based database (CastorEDC). A separate group of IBD patients without signs of liver disease was accrued from the WORK‐IBD study.^11^

Correlations between two different symptom scoring instruments were always based on data collected at the same time.

Data from the DILSTENT trial,^9^ in which different interventions during endoscopic retrograde cholangiography (ERC) in PSC patients were compared, were used to assess the ability of the SCCS to detect clinical change after treatment. In the DILSTENT trial 65 PSC patients with a dominant stricture based on imaging and/or clinical profile underwent ERC with balloon dilatation or stent placement. SCCS was scored at baseline and at 3 months after endoscopic treatment.

### 2.3 Validation process

The topics addressed in the FDA guideline for development and validation of PRO measures^12^ were followed. Face and content validity, the degree to which the questionnaire measures all relevant items for cholestatic complaints, was assessed by an expert panel with a clinician, researcher and methodologist and based on literature.

Internal consistency of the SCCS was quantified by Cronbach's‐α coefficient. Item‐item correlations, item‐total correlations and correlations after one‐item removal were calculated. As the SCCS measures different complaints instead of one single symptom the internal consistency is expected to be moderate (0.70‐0.85).^13^

Test‐retest reliability (reproducibility) was measured by issuing the SCCS twice with exactly 48 hours in between (T1 and T2). As the SCCS measures symptoms in the past 7 days, only minor changes in scores should be observed. The degree of reproducibility will be determined by the intraclass correlation coefficient (ICC). An ICC ≥ 0.7 is considered as good reproducibility.^14^

Criterion validity, defined as the correlation between an item and the gold standard instrument for that specific item, was evaluated. Scores of the widely accepted and validated questionnaires 5D itch scale,^15^ Fatigue Impact Scale (FIS)^16^ and the RUQ abdominal pain item from the Liver Disease Symptom Index (LDSI)^17^ were correlated to the pruritus, fatigue and RUQ abdominal pain items of the SCCS respectively. There is no gold standard questionnaire for fever, except for measuring body temperature. A correlation > 0.7 was considered as good criterion validity.^14^

Construct validity, consisting of convergent and discriminant validity, was determined using Spearman's Rho correlation. Convergent validity refers to the degree to which two constructs (ie SCCS pruritus item and 5D itch scale) that theoretically should be related, are indeed related. Discriminant validity on the other hand, refers to the degree to which two constructs that should not correlate are indeed unrelated. An expert panel a priori hypothesized direction and strength of the associations between SCCS and several validated questionnaires.^18^ Hypothesized correlations were compared to the observed correlations. Correlations from (−)0.3 up to (−)0.6 were considered moderate and (−)0.6 up to (−)1.0 were considered strong.

SCCS of patients from clinically different groups, such as Small Duct (SD)‐PSC vs Large Duct (LD)‐PSC were compared to determine known groups validity. SD‐PSC patients are expected to have lower SCCS as they are often asymptomatic. Conversely, patients with signs of end‐stage liver disease (varices, variceal bleed, ascites, hepatic encephalopathy and/or hepatorenal syndrome) are expected to have higher SCCS. Patients with impaired quality of life (based on EQ‐5D) or disease‐related absenteeism (based on IPCQ) are expected to have lower SCCS, while SCCS should be higher in liver transplanted patients. Differences in outcomes between (sub)groups of patients or within (sub)groups of patients over time were assessed after non‐parametric bootstrapping with 1000 simulations, drawing samples of the same size as the original samples and with replacement. Bias‐corrected and accelerated 95% confidence intervals are reported.

To assess if the presence of IBD would impact SCCS, scores of PSC patients with and without IBD were compared in all patients and subgroups of SD and LD patients. Moreover, PSC‐IBD patients were compared to IBD patients from a separate IBD cohort. To correct for difference in IBD activity patients were matched by propensity score matching based on IBD type (Ulcerative Colitis or Crohn's disease) and IBD activity (with a maximum disparity of 1 point on Harvey Bradshaw Index or Simple Clinical Colitis Activity Index). Statistical difference was tested by a paired *t* test. As multiple matching options were possible for some patients, eight alternative matching scenarios were run and pooled using Rubin's rules to account for within and between scenario variabilities.^19^

To assess the ability to detect clinical change (responsiveness) SCCS was measured before and 3 months after ERC in successfully treated patients(responders) and unsuccessfully treated patients (non‐responders) from the DILSTENT trial. Treatment response was determined either on biochemical response only, or on a combination of change in biochemical levels plus improve in SCCS. With regard to the cholestatic biochemistry criterion only treatment was considered successful if alkaline phosphatase (ALP) and/or bilirubin was decreased >30% after 3 months in patients with a baseline ALP and/or bilirubine of at least 1.2 × ULN. Patients with <20% decrease of ALP or bilirubin (if ALP resp. bilirubin was >1.2 × ULN at baseline) were considerd non‐responders. Change in SCCS item‐ and sum scores prior to and 3 months after ERC were measured with a paired *t* test following bootstrapping.

### 2.4 Adapted SCCS (SCCS‐A)—specification of severity and frequency

Some of the answer options of the SCCS contain a severity and a frequency domain. For example: the question about pruritus has the answer options ‘I have daily itch’ and ‘I have unbearable itch’. This might be confusing for patients with daily and unbearable itch. To evaluate whether this impacts the validity of the SCCS an adapted version with separate questions for severity and frequency was tested in parallel. This adapted SCCS (SCCS‐A) (Table S1) scores both severity (range 0‐4) and frequency (range 0‐4) of pruritus, fatigue and fever. A symptom score is the product of the frequency and severity score. For example, daily^3^ pretty much^2^ itch results in a pruritus score of 6. The SCCS‐A has the same time frame of 7 days as the original SCCS.

Patients’ experiences about both SCCS and SCCS‐A were scored. They were asked if they could express their level of symptoms better in one of the two questionnaires or whether there was no difference.

### 2.5 SCCS via a mobile application

The reproducibility of SCCS when sent via a mobile application was evaluated. Scores of patients who completed SCCS via both the digital questionnaire (by email) and the mobile application within 1 week were compared. Intraclass correlation coefficient (ICC) was evaluated to test the reproducibility. An ICC ≥ 0.7 was considered as good reproducibility.^14^

### 2.6 Other questionnaires

Individual items and the sum scores of SCCS and the SCCS‐A were compared to several validated PRO measures. A visual analogue scale (VAS) for pruritus (‘how much pruritus do you have at the moment?’), fatigue (‘How fatigued are you at the moment?’) and pain in the RUQ of the abdomen (‘How much pain do you have in the right upper quadrant of the abdomen at the moment?’) was used. The Dutch version of the EuroQol 5D‐5L (EQ‐5D‐5L) was used to measure general health status. For five domains (mobility, self‐care, daily activities, anxiety/depression and pain/discomfort) subjects rated the extent to which they experienced problems. This resulted in a health utility score ranging from −0.446 (worst) to 1.0 (best). Also, patients were asked to rate their general health score on a scale from 0 to 100. Pruritus was measured with the 5‐D itch scale.^15^ This score measures degree, direction, duration, disability and distribution of pruritus and results in a sum score ranging from 5 to 25. The FIS, a 40‐item questionnaire, was used to measure the degree of fatigue.^16^ Several domains of the LDSI 2.0^17^ were used to measure PSC‐related symptoms and impact of these symptoms on daily life.

Results were reported according to the CONSORT‐PRO Extension recommendations.^20^

## 3 RESULTS

### 3.1 Demographics

A total of 153 of 212 patients (72%) completed the questionnaire. Mean age of the responders was 54 years and median disease duration was 15 years (Table 2). Most patients had LD‐PSC (90%) and/or inflammatory bowel disease (56%).

**TABLE 2:** Demographics

Demographics	n = 153
Male gender (%)	93 (61)
Age (years, Mean ± SD)	54 ± 13
Disease duration in years (median (IQR))	15 (9)
Large Duct PSC (%)	104 (90)
Ursodeoxycholic acid use (%)	122 (81)
Signs of decompensated cirrhosis	14 (10)
Post‐liver transplant (%)	19 (12)
Inflammatory bowel disease (%)	64 (56)
Disease duration IBD (y), median (IQR)	20 (20.5)
Ulcerative colitis (%)	43 (67)
Crohns’ disease (%)	19 (30)
IBD‐U (%)	2 (3)
Biological use (%)	10 (7)
Harvey Bradshaw Index (median IQR))	1 (3.5)
Simple clinical colitis activity index (median IQR))	2 (4.3)

### 3.2 Score distribution

**FIGURE 1:**
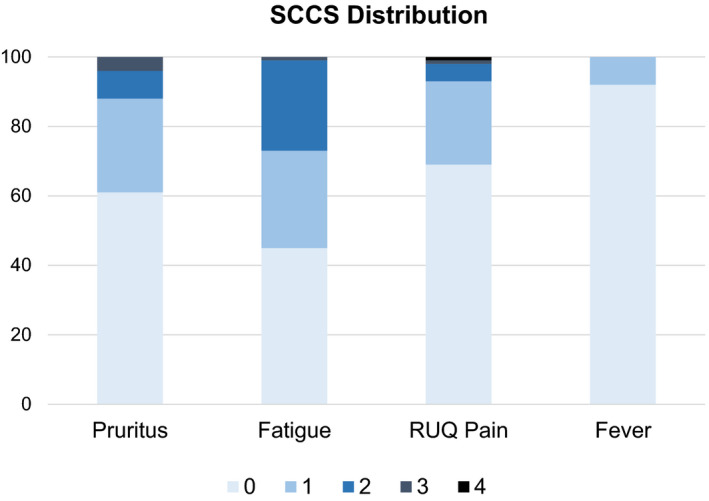
Percentage of patients with a SCCS score 0, 1, 2, 3 or 4 per item

Figure 1 shows the distribution of scores on the different SCCS questions. Mean scores on the pruritus, fatigue, RUQ abdominal pain and fever question were 0.55, 0.83, 0.40 and 0.08 respectively.

### 3.3 Validation

#### 3.3.1 Internal consistency

Item‐Item correlations are shown in Table 3. The pruritus, fatigue and RUQ abdominal pain item have moderate inter‐item correlations. The fever item only correlates moderately with RUQ abdominal pain. Item‐to‐total correlations from pruritus, fatigue, pain and fever are 0.50, 0.60, 0.53 and 0.30 respectively. Cronbach's‐α was 0.67. Removal of the fever item increased Cronbach's‐α to 0.71. Removal of other items decreased internal consistency.

**TABLE 3:** Internal consistency

	Fever item included			Fever item excluded		
	Item‐to‐item correlation	Item‐to‐total correlation	Cronbach's‐α if item deleted	Item‐to‐item correlation	Item‐to‐total correlation	Cronbach's‐α if item deleted
Pruritus	0.16‐0.51	0.49	0.58	0.34‐0.51	0.50	0.65
Fatigue	0.20‐0.51	0.60	0.50	0.49‐0.51	0.61	0.50
Pain	0.34‐0.49	0.52	0.56	0.34‐0.49	0.48	0.67
Fever	0.16‐0.39	0.30	0.71			

#### 3.3.2 Test‐retest reliability

A total of 57 patients filled in the SCCS twice with exactly 48 hours in between to measure reproducibility. The pruritus, fatigue, pain and fever score did not change in 82%, 88%, 88% and 100% of patients respectively. The sum score remained the same in 72% of all patients, 26% had a change of 1 point (4 patients −1 and 11 patients +1) and 1 patient had an increase of 2 points. Intraclass correlation coefficient (ICC) for pruritus, fatigue, RUQ pain and fever were excellent with 0.85, 0.93, 0.89 and 1.00 respectively. ICC for the sum score was 0.96.

#### 3.3.3 Criterion validity

The correlations between every SCCS item and its corresponding gold standard instrument were all high. Correlation between the SCCS pruritus item and the sum score of the 5D‐itch scale^15^ was 0.841, SCCS fatigue item and fatigue impact scale^16^ was 0.820 and RUQ‐A pain and the RUQ‐A pain item from LDSI^17^ was 0.833.

#### 3.3.4 Construct validity

The hypothesized and observed correlations between SCCS and relevant items of the LDSI, 5D‐itch score, EQ‐5D‐5L and VAS are presented in Table 4. Whether correlations were in line with a priori hypothesized correlations is indicated in the last column. Correlations between the SCCS pruritus item and convergent domains (VAS for itch, LDSI itch item and the 5D‐Itch score) ranged from 0.70 to 0.86, which is high. As expected, a negative correlation with EQ‐5D health index was found.

**TABLE 4:** A priori hypothesized and observed Spearman's Rho correlations of SCCS items and relevant domains of validated questionnaires

SCCS item	PRO instrument or domain	A priori hypothesized correlation	Observed correlation (95% CI)		Expectation ^a^
Pruritus	*VAS Itch*	0.6 to 0.9	0.70	(0.59; 0.79)	+
	*LDSI Itch*	0.6 to 0.9	0.86	(0.79; 0.92)	+
	*5D Itch score*	0.6 to 0.9	0.84	(0.75; 0.91)	+
	*EQ‐5D Index*	−0.6 to −0.3	−0.42	(−0.55; −0.28)	+
Fatigue	*VAS Fatigue*	0.6 to 0.9	0.77	(0.69; 0.85)	+
	LDSI Sleepiness	0.6 to 0.9	0.66	(0.54; 0.57)	+
	LDSI Sleep imp.	0.6 to 0.9	0.72	(0.62; 0.81)	+
	*EQ‐5D Index*	−0.6 to −0.3	−0.64	(−0.74; −0.53)	>
Pain	*VAS RUQ‐A pain*	0.6 to 0.9	0.67	(0.55; 0.78)	+
	*LDSI RUQ‐A pain*	0.6 to 0.9	0.83	(0.74; 0.15)	+
	*EQ‐5D Pain*	0.9 to 0.6	0.54	(0.41; 0.67)	<
	*EQ‐5D Index*	−0.6 to −0.3	−0.41	(−0.55; −0.27)	+
Fever	*EQ‐5D Pain*	0.6 to 0.3	0.26	(0.09; 0.83)	−
	*EQ‐5D Index*	−0.6 to −0.3	−0.14	(−0.28; 0.01)	−
Sum score	*EQ‐5D Health st*.	−0.9 to−0.6	−0.61	(−0.70; −0.90)	+
	*EQ‐5D Index*	−0.9 to −0.6	−0.64	(−0.74; −0.41)	+

Note

+correlation as expected.

<correlation weaker than expected.

>correlation stronger than expected.

−no correlation found, in contrast to expectations.

^a^ Final column indicates if the correlation was consistent with the a priori expectations of the expert panel:

The fatigue item correlated as expected with convergent domains (VAS for fatigue and LDSI sleepiness and sleepiness impact items). The correlation with EQ‐5D health index was stronger (−0.64) than expected. Correlations of the RUQ abdominal pain item with convergent items were as expected. The correlation with the pain and discomfort item of the EQ‐5D was weaker (0.54) than expected (0.60‐0.90). In contrast to the a priori hypotheses no correlations were found between the fever item and EQ‐5D pain and EQ‐5D index. A total of 12 of 16 correlations (75%) were in line with the predictions, 1 (6%) was a little higher than expected, 1 (6%) a little lower and for 2 items (14%) no correlation was found in contrast to expectations.

#### 3.3.5 Known groups validity

Mean SCCS sum scores from different groups were compared (Table 5). No significant difference was found between PSC patients with and without IBD. Small Duct PSC patients, who are in general considered to have a milder disease course, have lower SCCS compared to Large Duct PSC patients (0.83 vs 1.92). Substantial differences between LD‐PSC vs SD‐PSC patients were seen in both PSC only and PSC‐IBD patients (0.9 to 1.3) (Table S3). In addition, SCCS of PSC‐IBD vs PSC only were almost equal in both SD‐PSC and LD‐PSC (−0.2 to 0.2).

**TABLE 5:** 'Known groups' validity of SCCS

Group		n	SCCS sum score *mean (95% CI)*		Difference *Mean (95% CI)*	*P*‐value
IBD	*Yes*	65	1.88	(1.38;2.38)	0.23 (−0.40; 0.90)	.516
	*No*	51	1.65	(1.25;2.08)		
PSC type	*LD*	103	1.92	(1.59;2.27)	1.09 (0.21; 1.83)	.019
	SD	12	0.83	(0.42;1.33)		
End‐stage liver disease	*Yes*	14	3.71	(2.50;5.06)	2.02 (0.75; 3.44)	.003
	*No*	122	1.70	(1.40;2.00)		
Liver transplanted	*Yes*	19	1.00	(0.42;1.74)	‐0.99 (−1.67; −0.13)	.023
	*No*	134	1.99	(1.66;2.38)		
EQ‐5D Health score	*1*	98	0.40	(0.24;0.58)	‐2.17 (−2.57; ‐1.74)	.001
	*<1*	50	2.57	(2.17;2.94)		
Absenteeism	*Yes*	17	3.76	(2.99;4.65)	2.13 (1.18; 3.13)	.002
	*No*	136	1.63	(1.33;1.93)		

A total of 44 PSC‐IBD patients were matched to 44 IBD patients (Table S4). Scores on the pruritus and fever item were significantly higher in the PSC‐IBD group. SCCS sum score was on average 0.7 points higher in the PSC‐IBD group, however, with a *P*‐value of .054 this did not meet the criterion for significant difference.

In patients with clinical signs of end‐stage liver disease SCCS were higher. Patients with impaired general quality of life score, measured by EQ‐5D, have higher SCCS sum scores. Also, patients who cannot work because of disease (absenteeism) have higher SCCS.

#### 3.3.6 Detection of clinical change

In 41 patients who underwent ERC in the DILSTENT trial, treatment response could be rated on biochemical response only. This was considered succesfull in 27 patients according to the predefined criteria. The pruritus, fatigue and RUQ abdominal pain score of these patients decreased significantly 3 months after the intervention (Table 6). The intervention had no effect on the fever item, but this had a very low frequency (4/49) at baseline. Mean SCCS score dropped from 3.59 to 1.67 after treatment. No significant decrease of any item or the sum score was observed in the non‐responder group (n = 14).

**TABLE 6:** SCCS before and 3 mo after ERC in responders (n = 27) and non‐responders (n = 14)

SCCS domain	Prior to intervention *mean (95% CI)*	3 mo after intervention *mean (95% CI)*	Difference *mean (95% CI)*	*P‐value*
Responders (n = 27)				
Pruritus	1.59 (1.08; 2.15)	0.63 (0.31; 1.00)	−0.96 (−1.50; −0.41)	.002
Fatigue	1.15 (0.73; 1.55)	0.63 (0.30; 1.00)	−0.52 (−1.00; −0.09)	.039
RUQ‐A pain	0.74 (0.37; 1.13)	0.30 (0.10; 0.58)	−0.44 (−0.79; −0.04)	.046
Fever	0.11 (0.0; 0.22)	0.11 (0.0; 0.24)	0.0 (−0.17; 0.17)	1.000
Sum score	3.59 (2.70; 4.58)	1.67 (1.00; 2.48)	−1.93 (−2.87; −0.93)	.001
Non‐responders (n = 14)				
Pruritus	1.36 (0.69; 2.00)	0.79 (0.33; 1.31)	−0.57 (−1.33; 0.071)	.173
Fatigue	1.36 (0.87; 1.83)	1.00 (0.55; 1.50)	−0.36 (−1.00; 0.21)	.250
RUQ‐A pain	0.57 (0.20; 1.00)	0.36 (0.06; 0.73)	−0.21 (−0.67; 0.20)	.355
Fever				
Sum score	3.29 (2.00; 4.48)	2.14 (1.19; 3.17)	‐1.14 (‐2.54;‐0.052)	.109

^a^ One patient had fever at baseline and none of the patients had fever at 3 mo after the intervention; results not tested.

#### 3.3.7 Adapted SCCS with separate and frequency domains

Correlations of the SCCS‐A, with separate questions for severity and frequency, were compared to those of the original SCCS (Table S2). In general, correlations were very similar. The biggest difference in favour of SCCS was seen when comparing the pruritus item to LDSI itch (0.029). On the other hand, the biggest difference (0.060) in favour of SCCS‐A was seen when comparing to VAS for fatigue. The category of strength of the correlations was always the same for SCCS and SCCS‐A.

#### 3.3.8 Patients' experiences

A total of 47% of patients had no preference for SCCS or SCCS‐A, 23% of patients could express their symptoms better in SCCS and 30% preferred SCCS‐A.

#### 3.3.9 SCCS via a mobile application

A total of 69 patients completed the SCCS via email and the mobile application within 7 days (Table 7). Mean scores on the different items were highly comparable. ICC ranges from 0.65 to 0.92.

**TABLE 7:** SCCS item and sum scores via e‐mail and the mobile application (N = 69)

Item	E‐mail *mean (95% CI)*	Mobile app *mean (95% CI)*	ICC	95% CI
Pruritus	0.72 (0.55; 0.90)	0.72 (0.55; 0.90)	0.87	0.80; 0.92
Fatigue	0.91 (0.72; 1.09)	0.88 (0.70; 1.06)	0.91	0.86; 0.94
RUQ‐A pain	0.36 (0.25; 0.48)	0.33 (0.22; 0.48)	0.79	0.69; 0.87
Fever	0.07 (0.03; 0.12)	0.06 (0.01; 0.10)	0.65	0.49; 0.77
SCCS sum score	2.07 (1.65; 2.49)	2.00 (1.61; 2.41)	0.92	0.87; 0.95

## 4 DISCUSSION

In this study using FDA guidelines for development and validation of PRO measures we demonstrated that the quick and easy SCCS is a valid and reliable instrument to measure cholestatic symptoms in PSC patients. The SCCS shows good internal consistency, reproducibility, content‐, criterion‐ and construct validity as well as the ability to detect change after endoscopic treatment. Internal consistency, described by Cronbach's‐α was 0.708 without the fever item. Although there are no clear cut‐off values for Cronbach's‐α, in general a value > 0.7 is considered as ‘acceptable’ consistency.^13^ As expected, internal consistency was moderate because SCCS measures different cholestatic symptoms, which do not need to concur at any moment in time. In general, minor changes in scores were observed when assessing test–retest reliability. The high ICCs show that short‐term reproducibility of the SCCS is very good. Criterion validity was excellent for pruritus, fatigue and RUQ abdominal pain, which proves that these items exactly measure what they should measure. With regard to construct validity, correlations between SCCS items and relevant domains of other questionnaires were moderate to high except for fever. Almost all correlations (75%) were as hypothesized and no unexpected correlations were found. Unexpectedly, the fever item did not correlate with EQ‐5D pain/discomfort and general health status, but this may be attributable to the low frequency and aspecificity of fever. SCCS was able to differentiate between clinically different groups, but no difference was found between patients with and without IBD. The ability to detect change was clearly observed in succesfully treated patients. Scores of all items decreased after intervention in these patients. The fact that SCCS did not change significantly in non‐responders confirmes that SCCS is a valid tool to detect clinical change after intervention.

SCCS in PSC‐IBD patients is comparable to PSC only patients. The subgroups analysis comparing LD and SD patients with and without IBD revealed that PSC type had much more influence on scores than coexisting IBD. Severity of PSC, and not IBD, is the major driver of SCCS, hence the questionnaire is suitable for PSC only and PSC‐IBD patients. Moreover, PSC‐IBD patients had higher pruritus and fever scores compared to IBD only patients and a trend was observed on the sum score. Although all components of SCCS are typical PSC symptoms, they obviously are not specific for PSC alone. As IBD symptoms, in particular fatigue and RUQ abdominal pain, can be comparable to cholestatic symptoms, SCCS in patients with active IBD should be interpreted with some caution.

The adapted version of the SCCS, with separate questions for frequency and severity of symptoms, showed similar correlations with relevant domains as compared to SCCS. Moreover, most patients had no preference for either version. For that reason we do not consider the SCCS‐A superior.

The SCCS fever item decreased internal consistency, correlated poorly with other items, and did not change after intervention. For that reason we suggest that this item is not contributory to the SCCS and may be removed from the questionnaire. The small effect of the fever item is probably a result of its rare or transient occurrence.

Other PSC‐ or liver disease‐specific PRO measures such as PSC PRO and LDSI provide very detailed information about symptoms and disease burden.^6^, ^17^ However, they may be less suitable in clinical practice because of their length and complexity. Completing a PSC PRO questionnaire takes on average 7‐15 minutes.^6^ Filling in SCCS takes less than a minute, which makes it suitable for frequent issuing. Moreover, the capacity of the SCCS to detect clinical change, which is an important feature for use in studies, was not evaluated in PSC PRO and LDSI.

We have also evaluated issuing SCCS via a mobile application. Reproducibility when issuing SCCS via a mobile application compared to email was good and it was highly valued by both patients and researchers. One of the biggest benefits is that symptoms can be measured in daily life and home setting instead of only during hospital visits. This avoids recall bias and leads to more accurate and objective measurement of symptoms and disease burden.

A limitation of this study was that the population consisted of Dutch patients only. A next step will be to translate and validate the SCCS in other languages and populations, for instance through the newly formed International PSC Registry. Also, the added value of weighting items differently with regard to the sum score should be evaluated in a future study. Another limitation of this study is that at the time of data collection there was insufficient data available about the Child‐Pugh‐Turcotte stage to correlate the SCCS with this robust clinical disease staging score. Future studies should evaluate the validity of SCCS in relation to established measures of end‐stage disease. As no patients in this study had a cholangiocarcinoma at the time of issuing the questionnaire we cannot conclude that SCCS is a valid instrument to measure cholestatic symptoms of PSC patients suffering from cholangiocarcinoma. In general, these patients will have more severe symptoms leading to higher SCCS. We expect that these patients are highly comparable to patients with severe PSC without cholangiocarcinoma but cannot conclude that from the present data. Lastly, validity of the SCCS was tested using the FDA guidance for PROMs for clinical trials. Hence, although patients reported that the SCCS was very easy to complete either via a link prompted by email or by a mobile app, it was not formally fully validated for use in daily clinical practice.

In conclusion, we have shown that the SCCS is a valid PRO instrument for measuring cholestatic symptoms in PSC patients in clinical trials. It is quick, easy to use and responsive to change, which makes it particularly suitable for frequent issuing.

## CONFLICT OF INTEREST

All authors disclosed no financial relationship relevant to this publication.

## PATIENT CONSENT STATEMENT

All patients provided written consent for participation in this study.

## ETHICS APPROVAL STATEMENT

This study was approved by the IRB of the UMC Utrecht.

## Supporting information

Table S1‐S4
