# Supplementary material for: The Simple Cholestatic Complaints Score is a valid and quick patient‐reported outcome measure in primary sclerosing cholangitis
Source: Liver Int. 2020 Oct 28;40(11):2758–66. doi: 10.1111/liv.14644 (PMC7702029; doi:10.1111/liv.14644)
Supplement: Supplementary file 1 — Table S1‐S4 [file LIV-40-2758-s001.docx]

SUPPLEMENTARY Table 1. Scoring of the adapted Simple Cholestatic Complaints Score

| ***Score*** | ***Frequency*** | ***Score*** | ***Severity*** |
| --- | --- | --- | --- |
| *0* | Never | *0* | Nothing |
| *1* | Sometimes | *1* | A bit |
| *2* | Often | *2* | Pretty much |
| *3* | Daily | *3* | A lot |
| *4* | Continuous | *4* | Unbearable |

SUPPLEMENTARY Table 2. Absolute correlations of SCCS and SCCS-A and relevant questionnaires and difference in strength of correlation.

| **Item** | **Other PRO** | **SCCS** | **SCCS-A** | **Difference** |
| --- | --- | --- | --- | --- |
| Pruritus | VAS (itch) | 0.699 | 0.717 | -0.018 |
|  | LDSI Itch | 0.860 | 0.831 | **0.029** |
|  | 5D-itch score | 0.841 | 0.860 | -0.019 |
|  | EQ-5D Utility index | 0.418 | 0.395 | 0.023 |
| Fatigue | VAS (fatigue) | 0.772 | 0.832 | **-0.060** |
|  | LDSI Sleepiness | 0.658 | 0.675 | -0.017 |
|  | LDSI Sleepiness imp. | 0.719 | 0.711 | 0.008 |
|  | EQ-5D Utility index | 0.641 | 0.628 | 0.013 |
| Pain | VAS (RUQ-A pain) | 0.665 | 0.682 | -0.017 |
|  | LDSI RUQ-A pain | 0.833 | 0.835 | -0.002 |
|  | EQ-5D Pain | 0.540 | 0.534 | 0.006 |
|  | EQ-5D Utility index | 0.412 | 0.402 | 0.010 |
| Sum score | EQ-5D Health status | 0.605 | 0.654 | -0.049 |
|  | EQ-5D Utility index | 0.644 | 0.657 | -0.013 |

**SUPPLEMENTARY Table 3. SCCS in subgroups**

|  |  | **n** | **SCCS Sum score** *mean (95% CI)* | | **Difference** *mean*  *(95% CI)* |
| --- | --- | --- | --- | --- | --- |
| PSC-IBD | LD | 57 | 1.98 | (1.47; 249) | 1.32  (0.67; 2.04) |
|  | SD | 3 | 0.67 | (0.33; 1.00) |  |
| PSC only | LD | 41 | 1.80 | (1.32; 2.27) | 0.92  (-0.05; 1.79) |
|  | SD | 9 | 0.89 | (0.22; 1.56) |  |
| LD-PSC | *IBD* | 57 | 1.98 | (1.47; 2.53) | 0.18  (-0.63; 1.00) |
|  | *No IBD* | 41 | 1.80 | (1.29; 2.34) |  |
| SD-PSC | *IBD* | 3 | 0.67 | (0.33; 1.00) | -0.22  (-1.22; 0.56) |
|  | *No IBD* | 9 | 0.89 | (0.33; 1.44) |  |

**SUPPLEMENTARY Table 4. Mean SCCS and mean difference (95%CI) between PSC-IBD and IBD based on multiple matched datasets.**

| **SCCS Domain** | **PSC-IBD**  *mean*  *(95% CI)* | **IBD**  *mean*  *(95% CI)* | **Difference**  *mean*  *(95% CI)* | **P-value** |
| --- | --- | --- | --- | --- |
| Pruritus | 0.66  (0.39; 0.93) | 0.26  (0.11; 0.42) | 0.40  (0.10; 0.70) | 0.011 |
| Fatigue | 0.92  (0.64; 1.2) | 0.86  (0.59; 1.13) | 0.06  (-0.30; 0.42) | 0.743 |
| RUQ-A pain | 0.36  (0.16; 0.56) | 0.22  (0.09; 0.36) | 0.13  (-0.1; 0.37) | 0.263 |
| Fever | 0.11  (0.02; 0.21) | 0.02  (0.00; 0.07) | 0.09  (0.00; 0.17) | 0.046 |
| Sum score | 2.05  (1.41; 2.68) | 1.37  (0.96; 1.78) | 0.68  (-0.01; 1.36) | 0.054 |
